# Cancer Stem Cells and Glioblastoma: Time for Innovative Biomarkers of Radio-Resistance?

**DOI:** 10.3390/biology12101295

**Published:** 2023-09-28

**Authors:** Francesco Pasqualetti, Mario Miniati, Alessandra Gonnelli, Giovanni Gadducci, Noemi Giannini, Laura Palagini, Maricia Mancino, Taiusha Fuentes, Fabiola Paiar

**Affiliations:** 1 Radiation Oncology Unit, Azienda Ospedaliero-Universitaria Pisana, Via Roma 67, 56100 Pisa, Italy; francep24@hotmail.com (F.P.); gonnelli.alessandra@gmail.com (A.G.); ggadducci2@gmail.com (G.G.); noemi.giannini@yahoo.com (N.G.); ma.mancino@ao-pisa.toscana.it (M.M.); taiusha.fuentes@gmail.com (T.F.); fabiola.paiar@unipi.it (F.P.); 2 Department of Clinical and Experimental Medicine, University of Pisa, Italy, Via Roma 67, 56100 Pisa, Italy; lpalagini@gmail.com

**Keywords:** glioblastoma, radioresistance, cancer stem cells, biomarkers

## Abstract

**Simple Summary:**

Glioblastoma (GB) is characterized by a high level of resistance to radiotherapy. Recent studies focused attention on the importance of cancer stem cells (CSC) as elements that influence not only the growth of GB but also its resistance to available treatments. Therefore, stem cells’ radiosensitivity has become an important area of investigation. In this context, biomarkers of stemness (namely, the ability of a cell to perpetuate its lineage, give rise to differentiated cells, and interact with its environment to maintain a balance between quiescence, proliferation, and regeneration) seem to play an important role. The aim of this review is to summarize findings both on stem cells’ radiosensitivity and on biomarkers of stemness, in order to evaluate their relevance in this field.

**Abstract:**

Despite countless papers in the field of radioresistance, researchers are still far from clearly understanding the mechanisms triggered in glioblastoma. Cancer stem cells (CSC) are important to the growth and spread of cancer, according to many studies. In addition, more recently, it has been suggested that CSCs have an impact on glioblastoma patients’ prognosis, tumor aggressiveness, and treatment outcomes. In reviewing this new area of biology, we will provide a summary of the most recent research on CSCs and their role in the response to radio-chemotherapy in GB. In this review, we will examine the radiosensitivity of stem cells. Moreover, we summarize the current knowledge of the biomarkers of stemness and evaluate their potential function in the study of radiosensitivity.

## 1. Introduction

Glioblastomas (GB) are the most common primary malignant tumors of the central nervous system (CNS) [1]. Due to GB aggressiveness and resistance to radio-chemotherapy treatment (RTCT), the average survival of patients is just slightly longer than one year [2]. Radiotherapy in combination with temozolomide has been the standard postoperative therapy since the success of a phase 3 study led by Stupp in 2005 [2]. Unfortunately, there have been no improvements in the treatment of this tumor in the last 20 years, and all phase 3 studies have failed in their intention to change the standard therapy [3,4]. To date, the possible causes of underlying resistance to RTCT treatments are still not well understood, and the development of biomarkers of radio- and chemoresistance that can guide the development of new therapies is still ongoing [5,6,7]. Among the mechanisms involved in the failure of GB control and the aggressiveness of its recurrence, in the last decade, cancer stem cells are the subject of numerous studies [8]. In this descriptive review, we will evaluate the role of cancer stem cells in influencing the biology, in the resistance to radiotherapy, and in the behavior of extracranial solid cancers, focusing our attention mainly on glioblastoma [9]. Finally, we will discuss the development of new biomarkers related to them.

### 1.1. Cancer Stem Cells

Cancer stem cells (CSCs) are derived from normal stem cells and/or progenitor cells through genetic mutations and epigenetic changes. They represent a distinct subpopulation of cancer cells with unique characteristics including self-renewal, unlimited division potential, and the ability to differentiate in multiple directions. Initial detection of CSCs was reported in leukemia in 1997 and, subsequently, in various solid tumors such as brain, breast, colon, liver, and lung cancer [10,11,12,13,14,15]. CSCs are typically identified based on their ability to form tumors in immunodeficient mice. Similar to normal pluripotent stem cells, CSCs are long-lived and are able to remain quiescent in a dormant state [16]. They exert their control over various cancer characteristics (for instance, the ability to resist radio and chemotherapy) by interacting with tumor cells and the extracellular matrix (ECM) in their surrounding environment [17]. The genetic background of gliomas, including mutations such as IDH (Isocitrate Dehydrogenase) mutations, provides important insights into the behavior, classification, and potential biomarkers of these brain tumors. For example, IDH mutations, commonly found in lower-grade gliomas, have been a significant rea of study due to their impact on glioma biology and patient outcomes [18]. Glioma stem cells harboring IDH mutations evince molecular and genetic features relative to non-mutated glioblastoma stem cells (GSCs). These variances can exert influence upon cellular proliferation, viability, and differentiation pathways [19]. Moreover, IDH mutant gliomas manifest metabolic transformations; for example, the mutated IDH enzyme engenders an oncometabolite denoted as 2-hydroxyglutarate (2-HG), which is capable of impinging upon cellular processes and contributing to tumorigenesis [20].

Extracellular vesicles, including exosomes, as well as soluble factors such as interleukins, cytokines, and other metabolites are found in the tumor microenvironment (TME) [21,22,23]. These factors contribute to the establishment of a specialized tumor niche by attracting stromal cells, modulating angiogenesis, promoting metastasis, conferring resistance to anti-tumor treatments, and maintaining CSCs themselves through the secretion of specific molecules like IL-6, VEGF, and TGF-ß [24,25,26]. In this way they are responsible for inducing angiogenesis, displaying resistance to apoptosis, and exhibiting self-renewal and differentiation capabilities [27]. These properties suggest that CSCs could play a crucial role in tumor initiation, progression, metastasis, and treatment resistance [28]. For example, concerning radiosensitivity of gliomas, CSC-like cells contribute to radiotherapy resistance through activation of DNA damage response pathways and increased DNA repair capacity [29]. Similarly, in breast and colorectal cancer, CSCs are responsible for resistance to chemotherapy [30]. Moreover, the presence of CSCs in colon, breast, and brain tumors correlates with increased tumorigenicity and metastatic potential [31,32]. Nevertheless, the identification of these specific subpopulations has been challenging, and despite the description of various markers, the complexity of tumor heterogeneity and inter-patient variability makes it difficult to establish reliable and consistent markers [27]. Although the precise mechanisms underlying the pathogenic effects of CSCs are not fully understood, it is widely accepted that both intrinsic and extrinsic factors, along with mutations and epigenetic regulations, are primarily responsible for the development of CSCs involved in tumor initiation and progression. CSCs account for only a small proportion of the total tumor cell population (0.05–1%) within a tumor mass, which consists of a heterogeneous collection of tumor cells within the tumor microenvironment [28,33]. These CSCs that exhibit the expression of genes associated with stem cell markers, such as Oct4, Sox2, Nanog, c-kit, ABCG2, and ALDH, possess the capacity for self-renewal, leading to uncontrolled expansion of differentiated cell populations with altered molecular and cellular phenotypes. Ultimately, this contributes to the heterogeneity of primary and metastatic tumor cells within a tumor mass, which can display resistance to therapeutic interventions and contribute to tumor recurrence [34]. The discovery and characterization of CSCs in malignant diseases offer valuable insights into potential strategies for targeted inhibition or elimination of CSCs as a therapeutic approach to combat aggressive tumor phenotypes. Various stem cell markers have been identified in CSC populations isolated from diverse malignant diseases. Therefore, the identification of specific markers for CSCs, the isolation and characterization of CSCs from cancerous tissues, and the development of targeted approaches to eradicate CSCs present a promising avenue for advancements in cancer research.

Currently, the study of the role of CSCs in the radioresistance of gliomas is mainly limited to preclinical investigations. Moreover, with due limitations related to patient selection, static data, and partial representation of the tumor as a whole, to date, the translational development of biomarkers of CSCs in GB is linked to tumor tissue collected during surgery [7]. There is no exhaustive experience with the study of circulating biomarkers in CSCs [35].

### 1.2. Radiobiology of CSCs

Radiotherapy (RT) induces DNA damage, either directly or indirectly, through the generation of water-derived radicals and reactive oxygen species (ROS), which interact with macromolecules, lipids, and proteins [36,37,38]. Despite high rates of local tumor control, a significant rate of treatment failure remains a major limitation in radiation oncology [39]. Because local recurrence is often followed by a fatal metastatic spread, insufficient response to radiation (i.e., radiation resistance) represents a key driver of locoregional or distant recurrence and, therefore, affects patients’ prognosis. Recently, increasing evidence demonstrating the presence of multiple genetically diverse clones within various types of tumors, including GB, has been reported [40]. Moreover, not all cells constituting a tumor exhibit equal sensitivity to RT, including cancer stem cells (CSCs), which have been shown to be resistant to conventional treatments, including ionizing radiation [41,42]. On these bases, understanding the diverse radiosensitivity of different tumor cell subpopulations, particularly CSCs, is of great importance. The so-called “5Rs” or radiobiological determinants (Repair, Redistribution, Repopulation, Reoxygenation, and Radiosensitivity) influence the relative biological effectiveness of radiation in conventional radiotherapy (RT) [43]. CSC radiation resistance is given by the impact on these five factors, resulting in recurrence of the tumor and/or distant metastases [44,45,46,47]. Radiation therapy is known to induce cancer cells’ death mainly through direct or indirect DNA damage. CSCs, on the other hand, have certain intracellular molecular properties that help them avoid the cytotoxic effects of treatments, such as a high-efficiency DNA damage detection system and a better ability to fix damage. In fact, it is generally believed that CSCs are characterized by a significant enhancement of DNA repair mechanisms compared to their non-tumorigenic counterparts (NTC) [48,49,50]. This is linked to the induction of checkpoint-pathways in response to RT, resulting in a postponed cell cycle advancement that allows for the restoration of DNA damage [51,52,53,54]. Remarkably, CSCs have been found to repair DNA damage using a more precise and error-free process, homologous recombination (HR), rather than non-homologous end-joining (NHEJ) [55,56]. Regarding the redistribution of cancer cells, several studies have highlighted a slower proliferative capacity of CSCs (quiescent or latent cells) compared to further differentiated tumor cells [57]. Since rapidly dividing cells are more sensitive to radiotherapy than quiescent cells, CSCs can survive and remain dormant for a variety of times, ranging from weeks to decades. Once CSCs resume their ability to self-renew, these cells can cause a recurrence [58]. Ionizing radiation repeatedly selects GB CSC clones with genetic changes that protect them from damage and allow them to keep functioning to maintain the tumor. [59]. In addition, RT has been found to induce non-CSC reprogramming with the acquisition of functional CSC features in order to account for cell loss in the stem cell pool in response to cellular injury [60,61]. Thus, CSCs radiation resistance is linked to both intrinsic and acquired pathways. The heterogeneity of CSCs appears to vary between different niches and as a function of oxygen tension. Thanks to the unlimited self-renewal capacity, with a physiological concentration of oxygen, CSCs have a role in tumor repopulation between the radiotherapy fractions [60,62]. In hypoxic settings, however, they persist as quiescent radioresistant CSCs. Furthermore, hypoxic regions are distinguished not only by the absence of oxygen, which results in low ROS levels but also by the overexpression of ROS scavengers [29]. The overproduction of ROS scavengers in CSCs, through genes such as superoxide dismutase, superoxide reductase, glutathione peroxidase, and catalase, defends them from ROS-induced injury, which may increase tumor radioresistance [63]. Furthermore, CSCs’ improved mitochondrial respiratory ability stops ROS overloading, resulting in low levels of ROS and preserving CSCs from RT-induced cell death [64]. Along this line, regardless of oxygen tension, it has been shown that CSCs are equipped with an intrinsic radioresistance related to the lower ROS production upon radiation compared to non-CSC [65]. Consistent with ROS being critical mediators of radiation-induced indirect cell killing, CSCs develop less DNA damage and are preferentially spared after irradiation compared to NTC.

### 1.3. Biomarkers Related to CSCs Radioresistance

**Figure 1:**
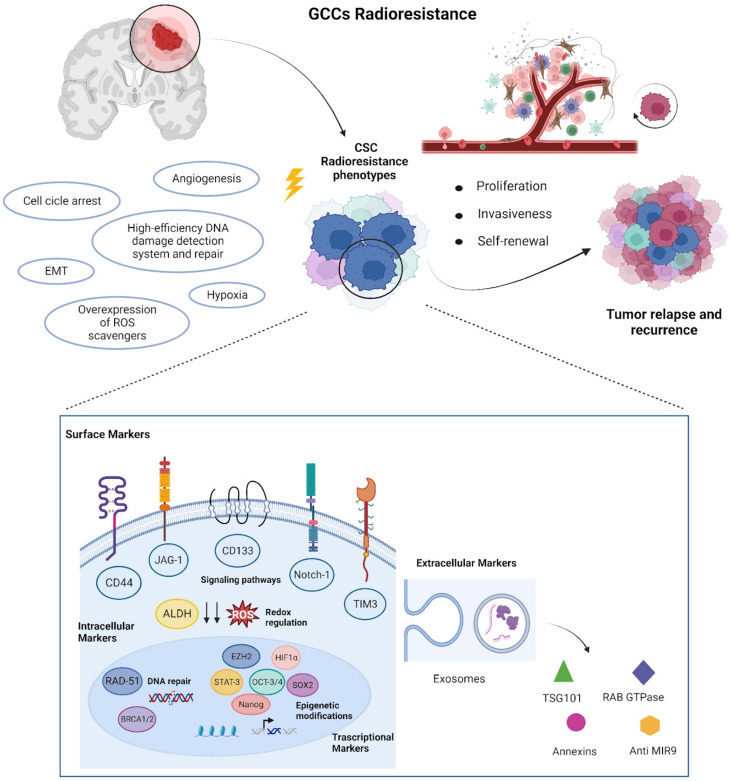
CSC-mediated mechanisms underlining radioresistance in GB. A high-efficiency DNA damage detection system, improved ability to repair DNA damage, overexpression of ROS scavengers, high plasticity associated to the epithelial mesenchymal transition process, capacity to maintain a quiescence state, neoangiogenesis, and hypoxia are factors that combine to create CSC radioresistant phenotypes. In particular, CSCs express biomarkers, which play a crucial role in radioresistance. These include cellular markers, such as surface, intracellular, and transcriptional, or extracellular factors, implicated in the activation of proliferation, self-renewal, and invasiveness pathways of CSCs: Surface Markers: CD44—cell surface adhesion receptor; CD133—prominin-1 transmembrane glycoprotein; Notch-1—notch homolog 1 translocation-associated receptor; JAG-1—jagged 1 Notch-1 ligand; TIM3—T cell immunoglobulin and mucin-domain containing-3 transmembrane protein; Intracellular Markers: ALDH—aldehyde dehydrogenases; RAD-51—recombinase; BRCA 1/2—breast cancer protein 1–2; Transcriptional Markers: EZH2—enhancer Of Zeste 2 Polycomb Repressive Complex 2 Subunit; OCT3/4—octamer-binding transcription factor 3–4; HIF—hypoxia-inducible factor; SOX2—SRY-Box Transcription Factor 2; Nanog—homeobox-containing transcription factor; STAT-3—signal transducer and activator of transcription 3; Extracellular Markers: TSG101—tumor susceptibility 101 protein; RAB GTPase—Ras-associated binding protein with GTPase fold; Annexins—calcium- and phospholipid-binding proteins; Anti-MIR9—anti microRNA9. ROS—reactive oxygen species. CSC—cancer stem cell. Created with BioRender.com, accessed on 16 September 2023.

In recent years, several stem cell-related biomarkers have been identified in various solid and hematological tumors, although clinical translation has not yet been achieved for the treatment of glioblastomas. Moreover, to make the development of stem cell-related clinical biomarkers even more difficult, similar transcription factors and signaling pathways are shared between CSCs and healthy stem cells. Listed below are reported the most important CSCs biomarkers that could play a role in radio-resistance in glioblastoma (Table 1 and Figure 1).

#### 1.3.1. Surface Markers

Membrane proteins known as the cluster of differentiation antigens (CD) play numerous roles in cell adhesion, communication, and differentiation.

- **CD 133**, also known as prominin-1, is a glycoprotein with five transmembrane regions first discovered as a hemopoietic cell biomarker. Chemoresistance has also been associated with the presence of CD133+ CSCs in oral cancer, lung cancer, and glioma (GB) (Table 1). Angelastro et al. propose that CD133 might contribute to the observed apoptosis resistance of CD133+ cancer progenitor cells. They demonstrate that ectopic overexpression of CD133 in rat C6 glioma cells results in significant resistance to camptothecin- and doxorubicin-induced apoptosis. Despite the fact that p53 was upregulated in CD133-overexpressing glioma cells treated with DNA-damaging agents, apoptosis appeared to be independent of p53. Tamura et al. obtained tumor samples from both the primary and secondary surgery of glioma 31 patients treated with postoperative RTCT [66]. The mean percentage of CD133-positive glioma cells in sections obtained during recurrence was 12.2% ± 10.3%, which was considerably higher than the percentage obtained during the initial surgery (1.08% ± 1.75%). The findings of the authors indicate that CD133-positive glioma stem cells can survive radiotherapy and chemotherapy, acquiring a proliferative cancer stem cell phenotype. This causes recurrence in de novo glioblastoma cases. Park et al. (2021) demonstrated that CD133 induces the PI3K/AKT-dependent activation of nuclear factor erythroid 2-related factor 2 (NRF2), an important transcription factor that protects the cell from ROS [92]. High NRF2 levels in spheroid-cultured HCT116 cells and the CD133-high subpopulation contributed to aggressive CSC phenotypes, such as anticancer radioresistance, sphere formation, anchorage-independent colony formation, and migration capability. Therefore, the NRF2 axis may be a promising target for inhibiting therapeutic radioresistance and enhancing survival capacity under stressful conditions in CD133-high CSCs.

Bao et al. showed that the activation checkpoint kinases Chk1 and Chk2 help CD133+ glioma CSCs make gliomas less sensitive to radiation [93]. Therefore, CD133-positive cells represent the cellular population that confers glioma radioresistance and may be the origin of tumor recurrence after radiation treatment. The researchers found that CD133+ tumor cells derived from human glioma xenografts and glioblastoma tissue collected after surgery trigger the DNA damage checkpoint more efficiently than CD133-negative tumor cells. Moreover, a specific inhibitor of the Chk1 and Chk2 checkpoint kinases may restore the radioresistance of CD133-positive glioma stem cells.

- The cell surface glycoprotein **CD44** is involved in cell adhesion, migration, and interaction [94]. CD44 plays a major role in both tumor neoangiogenesis and progression because of its affinity for messengers, such as growth hormones present in the tumor microenvironment as well as extracellular matrix elements including hyaluronan (HA) and osteopontin (OPN) [95]. According to a growing body of research, HA-CD44 interaction in the extracellular domain activates a number of signaling pathways, such as receptor tyrosine kinases (ErbB2 and EGFR) and transforming growth factor-receptor type 1 (TGF-R1) [96,97]. The study by Si et al. involving 62 patients with GB indicates that high CD44 expression is an indicator of a poor prognosis for GB patients. The median survival periods for those with high and low CD44 expressions were 3.5 and 18.5 months, respectively [67]. Liu et al. discovered, by analyzing primary cell lines obtained from GB patients, that CD44 is more abundantly present in radioresistant cells and acts a crucial role in stemness, cell proliferation, and angiogenesis. In fact, CD44+ cells expressed higher levels of ATM, Rad51, and CHK2 kinase phosphorilation compared to CD44-. These proteins are part of DNA damage response signaling pathway that is activated in response to cellular radiation damage [98].
- **TIM-3** (T-cell immunoglobulin mucin-3) is a type 1 cell-surface glycoprotein known to be expressed on the surface of leukemic stem cells and in more than 70% of patients with GB [68]. Through interaction with its ligand Galectin-9, TIM-3 causes aberrant catenin accumulation and constitutive activation of the canonical Wnt pathway. This phenomenon permits the maintenance and enhancement of cancer stemness [47]. Zhang et al. examined Tim-3 expression and MGMT promoter methylation in 84 GBs [68]. Therein, 62 patients out of 84 (73.81%) demonstrated mesenchymal Tim-3 expression in GB tissues, which was classified as low 15.48% (13/84), moderate 7.14% (6/84), or strong 51.14% (4/84) expression. The tumors of 48 patients tested positive for MGMT promoter methylation, while the tumors of 36 individuals tested negative. Tim-3 expression and MGMT promoter methylation status were found to be an independent risk factor for survival in GB patients. Strong expression of Tim-3 in conjunction with an unmethylated MGMT promoter was substantially linked with shorter OS in each of the four categories (*p* 0.05). Patients with no or low Tim-3 expression experienced a median survival of 16.9 and 16.4 months, respectively, but those with high Tim-3 expression and MGMT promoter nonmethylation had a median survival of 7.6 months. The average survival time for patients with low Tim-3 expression and methylation of the MGMT promoter was 21.8 months.

#### 1.3.2. Intracellular Markers

**Nestin** (neuroepithelial stem cell protein) is an important intermediate filament constituent that the cellular cytoskeleton usually presents in neuronal stem cells [88]. Increasing experiences have shown that it is expressed in several types of cancer including high grade glioma. Ishiwata et al. studied nestin activity in A172, a human high-grade glioma cell line, and in KG-1-C, a human low-grade glioma cell line [89]. Inhibition of nestin in A172 cell lines induced a reduction of cell growth rate and an inhibition of cellular invasiveness. Conversely, nestin overexpression, induced through the nestin expression vector in KG-1-C cells, caused the opposite effects. Nestin has been implicated also in radiochemoresistance by Chen et al. who demonstrated that nestin+ glioma stem cells are responsible for sustaining long-term tumor growth by generating transient populations of intensely proliferative cells after the exposure to TMZ [90]. In 2008, Zhang et al. observed, through Immunohistochemical analysis, that nestin expression levels were correlated with higher glioma grade (*p* < 0.05) [99]. The lower expression was significantly correlated with a better OS (*p* < 0.05). Patients with Nestin+/CD133+ expression had the lowest survival rate (*p* < 0.01). This finding was also confirmed at multivariate analysis (*p* < 0.01). In 2016, Guadagno et al. obtained the same results [91]. Furthermore, recent studies reported that Nestin has also been involved in neoangiogenesis [100]. Calabrese et al. demonstrated that Nestin+ glioma stem cells’ interactions with endothelial cells are fundamental for stem cell self-renewal and angiogenesis. Moreover, a retrospective study that analyzed nestin-expressing cells in 102 patients with glioma observed that proliferating endothelial cells expressing nestin correlate to histological grade and clinical outcome [101]. Hambardzumyan et al. highlighted that nestin-positive CSCs localized to the perivascular niche in medulloblastoma exhibited radioresistance through the activation of the AKT/PI3K and p53 signaling pathways.

- The **Notch** pathway controls cell division, differentiation, proliferation, and death and plays a fundamental role in central nervous system development. Protein convertases at site 1 (S1) cleave Notch receptors after they are synthesized, controlling their trafficking and signaling function. The intensity and timing of Notch activity are regulated by posttranslational changes of the receptors and ligands to generate context-specific signals [69]. The expression Pattern of Notch Signaling in Glioblastoma has been widely studied in the recent years. Wang et al. demonstrated that the synthesis of SOX2 induced NOTCH1 expression with consequent increasing of the GSCs’ invasiveness, making it extremely difficult for radiotherapists to cover effectively the radiotherapy target [102]. On the other hand, inhibition of Notch signaling reduced GSCs’ affinity towards white matter tropism. Han et al. analyzed Notch1 using immunohistochemistry in 69 glioma tissue specimens and 8 normal brain tissue specimens [103]. Multivariate analysis revealed that Notch1 expression was an independent adverse prognostic factor for survival. The effect of NOTCH1 down regulation, investigated on two glioma cell lines (U87MG and U251), was correlated with the reduction of GB proliferation. Moreover, Notch1 downregulation affected clonogenicity of GB cells and increased the number of γH2AX foci at 30 min and 24 h after irradiation at the dose of 8 Gy enhancing radiosensitivity. Notch1 inhibition also reduced angiogenesis, VEGF, and the hypoxic response RT.
- There is evidence that JAG-1 is implicated in radioresistance. KIM et al. irradiated JAG1-depleted LN18 cells with 3 and 5 Gy. The results showed colony survival was significantly reduced compared to LN18 cells that normally expressed JAG1 [104]. Another interesting protein in the NOTCH pathway is Jagged1 (JAG1) that is able to promote glioma-initiating cells (GICs) in HGG. Several studies demonstrated that JAG1 staining was strongly expressed only in glioma tissue. Meanwhile, no evidence in nearby normal brain tissue were found [105,106]. Hai et al. observed that high expression of Jagged1 was correlated with poor prognosis [6]. It was also evidenced in vitro that Jagged1 improved the invasiveness of glioma cells through the stimulation of the Nf-kb pathway. This study also demonstrated in vivo that the suppression of Jagged 1 inhibited the tumorogenis of Glioma cells improving the OS of mice compared to the control group. According Jubb et al., high JAG1 expression is also associated with type I microvascular pattern (MVP) that is notoriously associated with poor PFS and OS [107,108]. This finding was later confirmed by X.X Qui et al., who reported the Jagged1 expression in tumor and endothelial cells (EC) was correlated at the multivariate analysis with shortened time to progression (*p* < 0.001 for TC, *p* < 0.001 for TC) and OS (*p* < 0.001 for TC, *p* < 0.001 for TC). Lastly, there is evidence that JAG1 is also implicated in radioresistance. Kim et al. irradiated JAG1-depleted LN18 cells with 3 and 5 Gy. The results showed colony survival was significantly reduced compared to LN18 cells that normally expressed JAG1 [104].
- **Aldehyde dehydrogenase** (**ALDH**) is a class of NAD(P)+-dependent enzymes that play an important role in the detoxification process catalyzing the oxidation of aldehyde substrates [109]. ALDH is implicated in several cellular pathways which contribute to cancer cells’ radio- and chemoresistance; for example, it is involved in retinoid as well as β-Catenin/Tcf signaling pathways, which have been related to the stemness of CSCs [110]. Furthermore, high ALDH activity is correlated with low ROS cellular concentration, suggesting a strong antioxidant activity [109]. In HGG glioblastoma CSCs (GCC), ALDH expression is correlated with expression of mesenchymal phenotype (MES) and radio-chemoresistance. Conversely, ALDH- GCC was correlated with a pro-neural phenotype (PN) and a better prognosis. Mao et al. analyzed the 40 specimens collected from high-grade glioma patients. Transcriptome array analyses showed that ALDH genes were the most significantly expressed with an higher glycolytic activity in Mes GSCs (*p* = 0.000315), compared with PN GSCs (*p* < 0.01) [85]. ALDH expression was also more expressed in HGG specimens compared to low-grade glioma or normal brain tissue. Moreover, this study showed that radiotherapy induces transition of PN GSCs into a Mes-like GSC phenotype (PMT) that is highly resistant to radiation treatment. At the same time, the inhibition of ALDH attenuates the transformation in the radiation-resistant phenotype of Mes GSCs. Another study analyzed 30 surgical specimens (*n* = 30) collected from adult patients with histopathologically confirmed diagnosis of GB. High ALDH mRNA expression was associated with the poorer OS (*p* < 0.01, HR = 3.170, 95% CI: 1.328–7.566) and higher grade of peritumoral edema compared to the low expression group. In addition, Wang et al. demonstrated that ALDH3B1 and ALDH16A1 affect proliferation and migration of HGG cells by inducing cell-cycle arrest and the epithelial–mesenchymal transition [86].
- Research into DNA repair pathways, particularly involving RAD51 and BRCA1/2, has shed light on their critical roles in glioblastoma. These pathways are central to maintaining genome stability and influencing the behavior of CSCs. A pivotal investigation conducted by Balbous et al. assessed the expression of RAD51 within glioblastoma stem-like cells and its consequential association with resistance to radiation [87]. This underscored the pronounced role of RAD51 in the realm of treatment resistance. Furthermore, RAD51 holds the potential for involvement in the perpetuation of glioblastoma stem-like cells, entities speculated to underlie tumor growth, recurrence, and resistance to therapeutic regimens. The prospective targeting of RAD51 could potentially disrupt the processes of self-renewal and survival within these stem-like cells.
- **STAT3** (Signal transducer and activator of transcription 3) has been reported to be permanently activated in a variety of tumors, including GB, resulting in raised radio-resistance [82]. Masliantsev et al. demonstrated in 2018 that inhibiting STAT3 prior to cell irradiation reduced the surviving fraction of CSCs, implying that this technique could amplify radiation effects [83]. In addition, they used clinical specimens to evaluate STAT3 activation status in 61 GB patients, discovering a preferential phosphorylation of STAT3 on Serine727 (pS727). Furthermore, the investigators discovered that pS727 was linked to a significantly worse overall patient survival and was free of progression time. Taken together, these findings imply that pS727-STAT3 could be a prognosis marker as well as a therapeutic target for sensitizing highly radioresistant GSCs. Sherry et al. discovered in 2009 that treating GB-SC with two chemically separate small molecule STAT3 DNA-binding inhibitors reduces cell growth and the generation of new neurospheres from single cells [84]. STAT3 governs the proliferation and regeneration of GB CSCs, suggesting that it could be a viable target.

#### 1.3.3. Transcription Factor as Biomarker

- Transcriptional regulator **Sox2** (SRY-Box Transcription Factor 2) is responsible for the maintenance of an undifferentiated cellular phenotype [111]. SOX 2 is also linked to resistance to anti-tumor therapy through SOX2-mediated activation of ABC transporters, which can efflux drugs across the cell membrane [112]. According to an in vitro study conducted by Wang et al., Sox2 induced the dedifferentiation of differentiated glioma cells cultured in 1% of O2 [73]. Other studies also showed that SOx2 is implicated in white matter GSC tropism and also in the development of temozolomide resistance [113].
- The HIF-1 (Hypoxia-Inducible Factor 1-alpha) pathway plays an important and nuanced function in glioblastoma, particularly in response to the neoplasm’s hypoxic condition [114]. Under hypoxia, HIF-1 can be persistently expressed and works as an essential molecule in regulating the production of CSCs; however, the exact method is still unknown [114]. HIF-1 has been linked to the development of CSC markers such as OCT4, SOX2, NANOG, and KrÃ1⁄4ppel-like factor 4 (KLF4) [70,71,115]. Furthermore, suppressing HIF-1 can slow tumor progression by reducing the production of CSC biomarkers. For instance, HIF-1 has been found to attach directly to the CD47 promoter, facilitating gene transcription, assisting in the avoidance of macrophage phagocytosis, and maintaining the stem phenotype of breast CSCs [71]. Activation of HIF-1 in response to a hypoxic condition activates several genes that may increase the radioresistance of irradiated tumors (reducing the response to treatment) and is a negative prognostic factor [72].
- **EZH2** (Enhancer of zeste 2 polycomb repressive complex 2 subunit) is a part of a multimeric proteic complex called PRC2 consisting of three other subunits termed EED, SUZ12, and RbAp46/4. EZH2 has also been implicated in radio-resistance. Kim et al. assessed the impact of ionizing radiation on three glioma sphere samples (GB83, GB1123, and GB528) and detected a significant increase in both mRNA expression and protein levels of the EZH2/MELK–FOXM1 axis [76]. The effect of radiation on mice xenografted with spheres of GB with EZH2/MELK–FOXM1 axis genes silenced. The mean OS was longer in these mice compared to that of the control animals with activated EZH2/MELK–FOXM1 axis genes. These data demonstrated that EZH2/MELK–FOXM1 axis protein upregulation could promote tumorigenesis in vivo models (162). Wang et al. also evidenced the role of EZH2 in radioresistance; the expression of NEK2 expression, a protein that protectsprotect EZH2 from ubiquitin degradation in GCSc, can induce radioresistence in animal models [116]. EZH2 catalyzes the trimethylation of H3K27 which is associated with transcriptional repression and heterochromatin formation. This enzyme has been associated also with poor prognosis in HGG. A preclinical study demonstrated that EZH2 expression is significantly upregulated in the U87 and U251 glioma cells compared to HA-1800 human astrocytes. In glioma tissues EZH2 expression is grade dependent. In fact, higher expression levels have been detected in GB cells. Finally, the analysis of the Chinese Glioma Genome Atlas (CGGA) data set revealed that patients in the high-EZH2 group had a worse prognosis compared to those in the low-EZH2 group [117]. A possible role of E2F7−EZH2 axis on AKT/mTOR activation through PTEN suppression emerged from in vitro and in vivo experimental models. Another study demonstrated that EZH2 promotes M2 macrophage polarization in HGG resulting in macrophage-dependent disease development [118]. EZH2 also enhances the surface NKG2D ligands suppression on NK cells thus preventing immune response against GCSc [74]. Furthermore, EZH2 enhances chemoresistance to TMZ through stabilization of PARP1 protein [119].

#### 1.3.4. Extracellular Biomarkers

Exosomes are extracellular vesicles (30–160 nm in size) released from different type of cells, including GB cells, and are one of the main cell-to-cell communication tools. In recent years, thanks to the development of bioinformatics and, thus, the possibility of studying the content of the exosomes produced by tumor cells using a computational approach, these vesicles have assumed an increasingly important role in the study of glioblastomas. Their contents in terms of DNA, mRNA, miRNA, other non-coding RNA, proteins, lipids, and metabolites play critical functions in cancer progression [88,89,90] regulation of oncogene expression, mediation of signaling pathways, remodeling of tumor related fibroblast, regulation of cell radiosensitivity, and so forth [120,121,122]. Genetic materials can be delivered via exosomes in order to regulate gene expression with protein and miRNA [123]. Tumor cells secrete a much higher number of exosomes than normal cells [90] and, in some cases, have been demonstrated to have a direct correlation between the exosomes quantity and malignancy [124]. The composition and quantity of exosomes, as well as their biological effect on recipient cells, are affected by cellular stress. Exosomes are a significant environmental factor for cellular stress, and radiation can increase the release of exosomes and affect exosome-based intercellular communication, as observed in numerous normal and tumor cell lines [125]. It has recently been shown that radiations change the composition of released exosomes in both tumor and normal cells and also increased the uptake of exosomes by cells [77]. These released exosomes are capable of transferring radio-induced effects to non-irradiated cancer cells and even distant tissues and organs (mediating radiation bystander effects) [77,126]. Recent experiences have revealed that there are discrepancies between exosomes derived from CSCs and those derived from other cells of GB. Exosomes derived from CSCs contain multiple proteins, such as TSG101, Rab GPTase, annexins, and signal transduction molecules, which may be associated with their biogenesis, targeting, and putative immunological function [127]. For instance, Munoz et al. demonstrated that CSC-derived exosomes could deliver anti-miR-9, which inhibits miR-9, involved in the expression of pgb (P-glycoprotein) and the inhibition of multidrug transporter, enhancing the response of GB cells to temozolomide [128]. Moreover, Dai et al. in 2019 demonstrated that exosomes derived from low-level AHIF GB cells suppressed viability, invasion, and radioresistance, whereas exosomes derived from AHIF-overexpressing GB cells promoted viability, invasion, and radioresistance [129]. Sun et al. conducted an intriguing investigation into the effects of CSC-produced exosomes on GB cells in 2020 [130]. Non-CSC glioma cells were treated with GB CSC-released exosomes in the assumption that these exosomes could alter the phenotype of all GB cells. The Notch1 signaling pathway was activated in GSCs; Notch1 protein was highly enriched in GSC exosomes; Notch1 signaling pathway and stemness-related protein expressions were increased in GSC exosome-treated, non-GSC glioma cells and tumor tissues generated by these cells. The results of this study indicated that CSC exosomes function as information carriers, mediated the dedifferentiation of non-GSC glioma cells into GSCs by delivering Notch1 protein via Notch1 signaling activation, and increased the stemness and tumorigenicity of non-GSC glioma cells.

## 2. Conclusions and Future Directions

One of the most important weapons in the treatment of glioblastomas (as well as other solid tumors) is represented by radiotherapy. Unfortunately, due to a series of aspects that are still poorly understood, the radio-sensitivity of these tumors is not satisfactory, and, to date, we have few biomarkers that can help identify the most appropriate approach able to overcome the radioresistance of GB. 

Over the past 20 years, despite the development of new radiotherapy techniques and several successes achieved in the treatment of various solid tumors, the survival of glioblastoma patients has remained essentially the same, just a little more than a year. In order to break the impasse, increasing evidence is showing that CSCs, a limited proportion of tumor cells, may play an important role in the failure of cancer therapies and, above all, in tumor progression following radiotherapy treatment. For that reason, the study of CSCs to fight the radioresistance exhibited by GB represents a pivotal research frontier with profound potential implications. Research conducted in preclinical models has demonstrated that radiation therapy has a selective effect on eliminating differentiated tumor cells while preserving cancer stem cells. Furthermore, it has been observed that radiation exposure induces the upregulation of certain genes, including SOX2, OCT4, and NANOG. These genes encode transcription factors that play a crucial role in the acquisition of a cancer stem cell phenotype by differentiated tumor cells.

As we reported in this review, there are several biomarkers related to CSCs that have been associated with an increased radio- and chemoresistance of glioblastoma cells. However, unfortunately, to date, there are few translational studies conducted on glioblastoma patients, and the available literature consists mainly of in vitro studies. Translational studies conducted on glioblastoma patients might be more useful in the real world, where GBs have a lot of genetic and phenotype variation. These studies could also look at the connection between cancer stem cells and the tumor microenvironment, which is becoming more and more important in discussions about why some therapies do not work. Further impulse for the characterization of stemness biomarkers will come from the introduction of bioinformatics in translational research. Studying the individual gene may give insight into its potential to limit the effectiveness of ionizing radiation; however, each gene must be embedded in a network of genes that are activated or suppressed in response to a stimulus or insult, making the application of bioinformatics essential to characterizing the role that multiple biomarkers of stemness may play together. 

In the end, the study of CSCs and the numerous biomarkers associated with them may enable researchers in the near future to plan prospective translational studies and identify radioresistance factors. This approach could provide researchers with targets we need to improve the activity of ionizing radiation and, at the same time, to be able to develop an innovative personalized therapy for each GB patient. 

## Figures and Tables

**Table 1:** Tumor stem cell-specific biomarkers involved in cancer biology and resistance to treatment.

Surface Biomarker	Biological Action	References
CD133	Antioxidant scavenger system CD133-induced hypoxia	[66]
CD44	CD44 interaction with extracellular domain activates a number of signaling pathways implicated in tumor angiogenesis, proliferation and stemness	[67]
TIM 3	Galectin-TIM-3 interaction causes canonical Wnt pathway and permits the maintenance and enhancement of cancer stemness	[68]
Notch-1 and Jagged-1	Promote CSC’s invasiveness and white matter tropism, proliferation, angiogenesisand glioma-initiating cells (GICs)	[69]
**Transcription Biomarker**		
NANOG	Induces suppression of differentiation and cellular stamness	[70,71,72]
SOX2	Increases white matter GSC tropisim, drug resistance, epithelial-mesenchymal transition andangiogenesis	[73]
OCT3/4	Drug efflux pump Increases invasiveness, migration and cell proliferation Induces tumor angiogenesis regulating the homologous recombination factors	[74,75]
EZH2	AKT/mTOR activation Epithelial–mesenchymal transition (EMT) Silences transcription through trimethylation of histone H3 lysine 27 Stabilizes DDB2 and promotes nucleotide excision repair	[74,76,77]
HIF1- alfa	Angiogenesis Metabolic reprogramming (Warburg effect) Increases the invasion of cancer cells Immune system suppression	[78,79,80,81]
STAT-3	Proliferation Immune evasion Therapy resistance through upregulation of DNA repair proteins	[82,83,84]
**Intracellular Biomarker**		
ALDH	Activates retinoid as well as β-Catenin/Tcf signaling pathways related to the stemness of CSCs Antioxidant activity Expression of mesenchymal phenotype (MES) Affect proliferation and migration of cells by inducing cell-cycle arrest and the epithelial-mesenchymal transition	[85,86]
RAD51 and BRCA 1-2	DNA repair through homologous recombination Perpetuation of CSCs Sensitivity to chemotherapy	[87]
Nestin	Enhances invasiveness Generates transient populations of intensely proliferative cells Angiogenesis	[88,89,90,91]

## Data Availability

No new data were created.
